# Trigeminocardiac Reflex by Mandibular Extension on Rat Pial Microcirculation: Role of Nitric Oxide

**DOI:** 10.1371/journal.pone.0115767

**Published:** 2014-12-31

**Authors:** Dominga Lapi, Giuseppe Federighi, M. Paola Fantozzi, Cristina del Seppia, Sergio Ghione, Antonio Colantuoni, Rossana Scuri

**Affiliations:** 1 Department of Clinical Medicine and Surgery, “Federico II” University Medical School, Naples, Italy; 2 Department of Translational Research on New Technologies in Medicine and Surgery, University of Pisa, Pisa, Italy; 3 Institute of Clinical Physiology, National Council of Research (CNR), Pisa, Italy; 4 Fondazione Toscana Gabriele Monasterio - Medical and Public Health Research, Pisa, Italy; VCU, United States of America

## Abstract

In the present study we have extended our previous findings about the effects of 10 minutes of passive mandibular extension in anesthetized Wistar rats. By prolonging the observation time to 3 hours, we showed that 10 minutes mandibular extension caused a significant reduction of the mean arterial blood pressure and heart rate respect to baseline values, which persisted up to 160 minutes after mandibular extension. These effects were accompanied by a characteristic biphasic response of pial arterioles: during mandibular extension, pial arterioles constricted and after mandibular extension dilated for the whole observation period. Interestingly, the administration of the opioid receptor antagonist naloxone abolished the vasoconstriction observed during mandibular extension, while the administration of N_ω_-Nitro-L-arginine methyl ester, a nitric oxide synthase inhibitor, abolished the vasodilation observed after mandibular extension. Either drug did not affect the reduction of mean arterial blood pressure and heart rate induced by mandibular extension. By qRT-PCR, we also showed that neuronal nitric oxide synthase gene expression was significantly increased compared with baseline conditions during and after mandibular extension and endothelial nitric oxide synthase gene expression markedly increased at 2 hours after mandibular extension. Finally, western blotting detected a significant increase in neuronal and endothelial nitric oxide synthase protein expression. In conclusion mandibular extension caused complex effects on pial microcirculation involving opioid receptor activation and nitric oxide release by both neurons and endothelial vascular cells at different times.

## Introduction

In a previous study we reported that young normal volunteers subjected to a submaximal passive mandibular extension (ME), obtained by means of a dilator applied for 10 minutes between the upper and lower incisor teeth, showed a prolonged reduction in blood pressure (by 12–11 mmHg) and heart rate (by about 13 bpm). These effects appeared after 10 minutes ME and persisted for the successive 80 minutes. It had been suggested that ME could stimulate the terminal nerve branches triggering these responses [1]. This suggestion has been supported by experimental and clinical observations over the last 20 years which have demonstrated that the facial area is an important source of powerful autonomic reflexes that involve different systems including the cardiovascular system. For example, the stimulation of trigeminal receptors innervating the nose and the nasal passages was found to provide an important drive for the initiation of the trigeminocardiac reflex (TCR). TCR causes changes in systemic blood pressure, heart rate and, in some cases, gastric hypermotility [2]–[8].

A procedure as simple as ME, therefore, by causing important cardiovascular effects by trigeminal nerve stimulation, could have significant applications, especially in the control of systemic pressure.

Studies in experimental models have shown that TCR not only determines cardiovascular changes, but also influences the cerebral circulation [9], [10]. The systemic pressure and the cerebral circulation are closely related, because the cerebral microcirculation tends to self-adjust in order to preserve the brain from changes in blood flow during variations of systemic blood pressure. In a previous study on the effects of ME in rats [11], we have demonstrated that animals submitted to 10 minutes ME exhibited a reduction in the mean arterial blood pressure (MABP) and heart rate (HR) following the stimulation of the peripheral branches of the trigeminal nerve. When this nerve was cut the effects on MABP and HR disappeared. The hypotensive and bradicardic actions persisted for 80 minutes after 10 minutes ME in line with the observations carried out in humans [1]. Furthermore, in cerebral microcirculation, the pial arteriolar diameter significantly decreased during ME; after ME a vasodilation occurred which persisted for an observation period of 80 minutes. Therefore, ME appears to cause a hypotensive response and a specific regulation of pial microcirculation, but the underlying molecular mechanisms are still unknown.

The aim of the present study was to prolong the observation time after ME in order to establish the duration of the hypotensive and bradicardic effects and to evaluate the molecular mechanisms involved in the regulation of pial microcirculation.

In particular, we assessed the role of nitric oxide (NO) because it is widely involved in cerebral blood flow regulation [12], [13]. NO production may be due to the activation of endothelial cells, neurons or nitrergic perivascular nerves. Endothelial NO synthase (eNOS) and neuronal NO synthase (nNOS) are involved in constitutive basal and stimulated NO production, and inducible NO synthase (iNOS) generates NO when induced by the inflammation [14], [15].

## Materials and Methods

### Animals

Male Wistar rats weighing 250–300 g (Harlan, Udine, Italy) were used. The animals were individually housed in stainless steel cages in a room with a natural light–dark cycle and constant temperature (24±1°C) with access to food and water *ad libitum*.

This study was carried out in strict accordance with the recommendations in the Guide for the Care and Use of Laboratory Animals of the National Institutes of Health. The protocol was approved by the Committee on the Ethics of Animal Experiments of the University of Pisa (Permit Number: 001 4896/2013). All surgery was performed under alpha-chloralose and urethane anesthesia, and all efforts were made to minimize suffering.

### Drugs

Unless otherwise stated, all chemicals and reagents were obtained from Sigma-Aldrich (St. Louis, MO, USA). Primers for qRT-PCR were obtained from MWG (Ebersberg, Germany).

Fluorescein isothiocyanate (FITC) bound to dextran (molecular weight 70 KDa), 50 mg/100 g body weight (b.w.) was dissolved in 0.5 ml of saline solution for intravenous administration.

N_ω_-Nitro-L-arginine methyl ester (L-NAME), 10 mg/Kg b.w., was dissolved in 0.3 ml of saline solution for intravenous infusion (for 3 minutes, 10 minutes before ME). For local application on the parietal cortex (for 10 minutes, 10 minutes before ME), 10 mg/Kg b.w. L-NAME were dissolved in 1 ml of artificial cerebrospinal fluid (aCSF in mM: KCl 2.5, MgCl_2_ 1.4, CaCl_2_ 2.5, NaCl 119.0, NaHCO_3_ 26.2, urea 6.7 and glucose 11.0).

Naloxone solutions were obtained dissolving 40 µg/Kg b.w. in 0.3 ml of saline solution and intravenously infused (for 3 minutes, 10 minutes before ME) [16].

### Animal preparation

The rats were anesthetized with intraperitoneal injection of alpha-chloralose (50 mg/Kg b.w.) plus urethane (600 mg/Kg b.w.) for induction and with urethane alone (100 mg/kg, i.v., every hour) for maintenance. They were tracheotomized, intubated and mechanically ventilated with room air and a supplemental mixture of O_2_/CO_2_ (end-tidal CO_2_ was continuously measured by a CO_2_ analyzer and a respirator was adjusted to maintain end-tidal CO_2_ from 4.5 to 5.0% and to keep arterial blood gas tension within the normal range).

A catheter was placed in the left femoral artery which permitted to measure the arterial blood pressure. Another catheter was placed in the right femoral vein and used for the injection of the fluorescent tracer (FITC), about every 120 minutes during the whole experiment, and of urethane, about every 60 minutes.

Rectal temperature was monitored and maintained at 37.0±0.5°C with a heating stereotaxic frame, where the rats were secured.

To observe the pial microcirculation, a closed cranial window (4×5 mm) was implanted above the left parietal cortex (posterior 1.5 mm to bregma; lateral, 3 mm to the midline) [17]. To prevent overheating of the cerebral cortex during drilling, cold saline solution was suffused on the skull. The dura mater was gently removed and a 150 µm-thick quartz microscope coverglass was sealed to the bone with dental cement. The window inflow and outflow were assured by two needles secured in the dental cement of the window so that the brain parenchyma was continuously superfused with aCSF. The rate of superfusion was 0.5 mL/min controlled by a peristaltic pump. During superfusion the intracranial pressure was maintained at 5±1 mmHg and measured by a pressure transducer connected to a computer.

Throughout all experiments the values of mean arterial blood pressure (MABP) and heart rate (HR) were continuously recorded. The diameter changes of pial arterioles were monitored for 1 minute every 5 minutes. MABP was recorded with a Viggo-Spectramed P10E2 transducer (Oxnard, CA, USA) connected to the catheter in the femoral artery and HR was monitored with a Gould Windograf recorder (model 13-6615-10S, Gould, OH, USA). Data were recorded and stored in a computer for off-line analyses.

**Figure 1:**
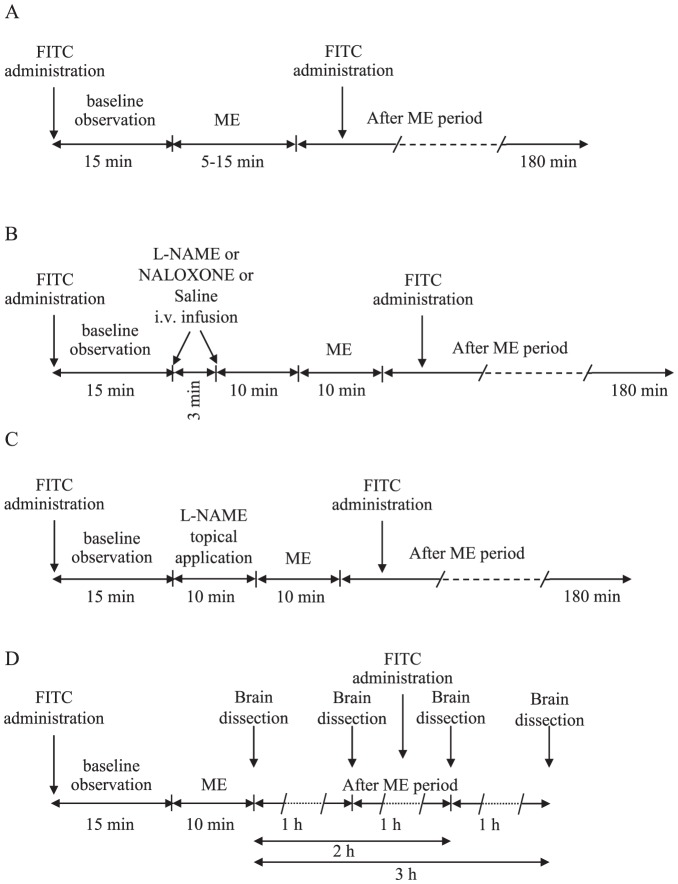
Outline of the different protocols used. (A) After 15 minutes of observation in baseline conditions, 5 or 10 or 15 minutes ME were performed and after removing ME post treatment measurements were carried out for 180 minutes. At the start of the experiment and about 100 minutes later, the fluorescent tracer was infused by the catheter placed in the right femoral vein. (B) After the 15 minutes baseline observation, L-NAME or naloxone or saline were i.v. infused by the catheter placed in the right femoral vein for 3 minutes. Then, after further 10 minutes in which the animal was kept in baseline conditions, ME was applied for 10 minutes. Afterwards post treatment measurements were carried out for 180 minutes. The FITC has been infused as in A. (C) 15 minutes of baseline observation were followed by a perfusion of L-NAME for 10 minutes in the cranial window open on the parietal region. Then, 10 minutes ME was performed followed by post treatment measurements for 180 minutes. The FITC has been infused as in A. (D) All animals were subjected to 15 minutes baseline observation and 10 minutes ME. At the end of the baseline period 3 rats were sacrificed for qRT-PCR and 3 rats for Western Blotting assays (Basal in Fig. 6D, 6E). Immediately after removing ME, 3 rats were sacrificed for qRT-PCR. Post treatment measurements were continued, and after 1 hour, 3 rats were sacrificed for qRT-PCR assay, after 2 hours 3 rats were sacrificed for qRT-PCR assay and 3 rats for Western Blotting assay, and after 3 hours further 3 rats were sacrificed for qRT-PCR assay. FITC was initially infused in all animals and every 100 minutes in the animals observed for prolonged times.

Figs. 1A, 1B and 1C report the different experimental protocols used to analyze the effects of a single ME (see below) on MABP, HR and the pial arteriolar diameter. Fig. 1D describes the experimental procedures carried out on rats whose brain were used in qRT-PCR and western blotting assays (see below).

The protocol in Fig. 1A permitted the evaluation of the effects of ME of different durations (5–15 minutes). Fig. 1B reports the protocol used to study the effects of L-NAME and naloxone intravenously administered. Control rats underwent the injection of saline solution and ME, or only the injection of L-NAME or naloxone. The protocol in Fig. 1C permitted studying the effects of the local application of L-NAME with or without ME.

In rats not subjected to ME [sham-operated (SO) rats], all parameters were monitored and recorded for 210 minutes.

Baseline measurements were obtained prior to ME (mean of 3 measurements at 5 minute intervals over 15 minutes) and were defined as baseline values (*B* in the Figures). Post-treatment measurements (AFTER ME in the Figures) were obtained immediately after ME (or, in the SO rats, 20 minutes after baseline observations) and were continued every 10 minutes for further 180 minutes.

### Mandibular extension

Mandibular extension (ME) was induced by a home-made U-shaped spring device placed between the two dental arches (superior and inferior) of the rat.

The spring device, appropriately designed for rats, consisted of two thin layers covered with a silicone elastomer (Sylgard, Dow Corning, Midland, MI) coupled to an adjustable spring.

The degree of ME was set at the upper limit of the opening of the mouth to avoid any muscle fatigue. Muscle fatigue was assessed in pilot experiments where the rats were subjected to different degrees of opening of the mouth; the tension of the masseter and anterior temporal muscles was evaluated by electromyography [18].

### Fluorescent microscopy technique and microvascular parameter assessment

Pial microcirculation was visualized with a fluorescence microscope (Leitz Orthoplan) fitted with long-distance objectives 2.5 x, numerical aperture (NA) 0.08, 10 x, NA 0.20, 20 x, NA 0.25, 32 x, NA 0.40, and a 10 x eyepiece and a filter block (Ploemopak, Leitz). Epi-illumination was provided by a 100 W mercury lamp using the appropriate filter for FITC and a heat filter (Leitz KG1). Pial microcirculation was televised with a DAGE MTI 300RC low-light level digital camera (2.1 megapixel) and recorded by a computer based frame grabber (Pinnacle DC 10 plus, Avid Technology, MA, USA).

Video images were videotaped and microvascular measurements (diameter and length) were made off-line using a computer-assisted imaging software system (MIP Image, CNR, Institute of Clinical Physiology, Pisa, Italy). The results of diameter measurements were in accordance with those obtained by the shearing method (±0.5 µm). To avoid bias due to single operator measurements, two independent “blinded” operators measured the vessel diameters. Their measurements overlapped in all cases.

The arteriolar network was mapped by stop-frame images and pial arterioles were classified according to a centripetal ordering scheme (Strahler method, modified according to diameter) [19]. Order 0 was assigned to capillaries; order 1 to terminal arterioles and progressively higher orders to the vessels upstream. When two vessels of the same order joined, the parent vessel was assigned the next higher order. If two daughter vessels were of different orders, the parent vessel retained the higher of the two orders. The procedure of the pial arterioles classification was previously reported [20]. Arteriolar diameters and capillary red blood cell (RBC) velocity were measured with a computer-assisted method (frame by frame) in an area of 750×750 µm and expressed as mm/s.

In each animal one order 5, one order 4, two order 3 and four order 2 arterioles were studied during each experiment. We chose to present only the data regarding order 2 vessels, the most numerous arterioles in each preparation.

### Quantitative real-time RT-PCR (qRT-PCR real time)

Parietal cortex was dissected from rats sacrificed during the baseline observation period or immediately after the end of 10 minutes ME, or 1, 2 or 3 hours after ME according to the protocol shown in Fig. 1D (see above). The brain samples were frozen in liquid nitrogen and stored at −80°C. Total RNA was extracted from three samples for each experimental condition using ClCs gradient in accordance with Chirgwin et al. [21]. The purified RNA was suspended in RNase-free water, quantified spectrophotometrically (SmartSpec 3000; Bio-Rad, Hercules, CA, USA), treated with Amplification Grade DNase I (Sigma-Aldrich) and reverse-transcribed into cDNA (500 ng to sample) by using iScript cDNA synthesis kit (BioRad, Hercules, CA, USA).

Quantitative real-time PCR (qRT-PCR) was performed with the SYBR Green kit on the MiniOpticon Two-Color Real-time PCR detection system (Bio-Rad, Hercules, CA, USA). The primer pairs used: nNOS, eNOS, iNOS and glyceraldehyde 3-phosphate dehydrogenase (G3PDH) were designed using the software Primer Express Software v3.0.1 (Applied Biosystems, Foster City, CA, USA). The forward and reverse primers were chosen to hybridize a single specific region of the appropriate gene sequence and are listed in Table 1.

**Table 1:** Primer sequences used in qRT-PCR assay.

Gene	Primer Sequences	Accession N°
nNOS	F:TCATCATCTCAGACCTGATTCGA R:TCTACCAAGGGGCGACCGT	NM_052799.1
eNOS	F:TGGATCTAGACACCCGGACAAC R:GTCACTTTGGCCAGCTGGTAA	NM_021838.2
iNOS	F:GAAAGCGGTGTTCTTTGCTTCT R:CGCTTCCGACTTTCCTGTCAA	NM_012611.3
G3PDH	F:GCTCTCTGCTCCTCCCTGTTC R:CGACCTTCACCATCTTGTCTATGA	NM_017008.4

For the PCR reaction 15 µL master-mix were prepared using 7.5 µL iQ Sybr Green Supermix (200 µM dNTP, 5 mM MgCl2, 3.75 U iTaq DNA polymerase), 0.5 µg/µl cDNA, 300 nM of each primer and RNase-free H_2_O. Cycling conditions included initial denaturation (3 minutes at 95°C), amplification, the quantification program repeated 40 times (10 sec at 95°C, 60 sec at 57°C with a single fluorescent measurement at the end of each elongation step) and the dissociation protocol (from 60°C to 95°C by 1°C increments followed by a 30 sec hold and fluorescent measurement).

According to the protocol indicated by Bio-Rad (Hercules, CA, USA), the primers' efficiency had been previously assessed (see Tab 1S). Each target gene was loaded with G3PDH, used as the reference gene. Before using it, we tested the transcriptional stability of G3PDH. In three biological replicates for each of which we did three technical replicates, we evaluated the Ct value in samples from rats sacrificed during the baseline condition, immediately after 10 minutes ME or 1, 2 or 3 hours after ME. The Ct values of G3PDH were comparable with each of the genes we analyzed in this work and showed small changes in the various samples (see Fig. S1 and Tab. S2 in S1 File).

The relative gene expression values (2^−ΔΔCt^) were calculated using G3PDH as the endogenous reference gene and samples from rats sacrificed during the baseline condition as the calibrator sample (control sample), following the calculation described by Livak et al. [22]. This method assumes that gene is amplified with an efficiency close to 100% and not less than 5%.

### Western Blotting

Two hours after ME, 3 rats subjected to ME and 3 rats which had been subjected only to 15 minutes baseline observation were sacrificed to obtain samples of parietal cortex areas. The tissues were homogenized in 300 µl of lysis buffer containing: 20 mM Hepes at pH 7.4, 1.0 mM NaN3, 1% Triton X, 0.2 mM Na3VO4, 50 mM NaF, and also a protease/phosphatase inhibitors cocktail. The homogenates obtained were centrifuged at 7.000 rpm for 40 minutes at 4°C, the supernatant was collected and the protein concentration was determined using the Lowry's method.

Supernatant containing total proteins was quantified and 80 µg of each sample were separated on 12% SDS–PAGE and transferred onto Hybond ECL nitrocellulose membranes (Amersham Pharmacia Biotech, USA) by electroblotting at 100 V for 60 minutes at 4°C in transfer buffer (25 mM Tris-HCl, 192 mM glycine, pH 8.3, 20% methanol). Subsequently, the membranes were subjected to blocking for 60 minutes at room temperature in TBS (Tris Buffered Saline) 1X, 0.1% Tween 20, 5% non-fat dry milk (Bio-Rad, Hercules, CA, USA) and then incubated overnight at 4°C with the primary antibody polyclonal anti-eNOS (Santa Cruz Biotechnology Inc., CA, USA) diluted 1∶500 in 1X TBS, 0.1% Tween 20 and 3% non-fat dry milk. After 3 washes in 0.1%, TBS-Tween, the membranes were incubated for 60 minutes at room temperature with the secondary antibody anti-rabbit IgG labeled with horseradish peroxidase (Amersham Pharmacia Biotech UK Limited), diluted 1∶2000 in 1X TBS, 0.1% Tween 20 and 3% non-fat dry milk. Finally other 3 washes were performed in 0.1% TBS-Tween.

The signals were detected by chemiluminescence with ECL (Amersham Pharmacia Biotech UK Limited). The relative intensity of protein bands was measured using the Molecular Imager Chemi-Doc imaging system (Bio-Rad, Hercules, CA, USA) and evaluated by the Quantity One software (Bio-Rad, Hercules, CA, USA).

The β-tubulin was used as an internal control and the optical density was normalized vs control and reported in the form of Arbitrary Units (AU).

### Statistical analysis

All data were expressed as mean ± S.E. Data were tested for normal distribution with the Kolmogorov-Smirnov test. Due to the normality of distribution, one way ANOVA for repeated measures was used for testing the changes in MABP, HR and pial arterioles diameter. For post-hoc analysis, the Dunnett's multiple comparison test was done.

The significance of changes in capillary RBC velocity was evaluated by one way ANOVA for repeated measures when three values needed to be compared or by the paired *t* test when only two values were considered.

The increase or decrease of gene expression at different times after ME was determined by comparing the target gene in rats sacrificed immediately after 10 minutes ME or 1, 2 or 3 hours after ME. and in control rats (sacrificed during the baseline condition) with a one way ANOVA design after normalization to G3PDH. All reactions were made in triplicate and data were plotted in histograms as mean ± E.S.

The unpaired *t* test was made to evaluate the significant differences in protein expression.

The statistical analysis was carried out with SPSS Statistics 20 package. Statistical significance was set at p<0.05.

## Results

### Effects of ME on mean arterial blood pressure (MABP), heart rate (HR) and pial microcirculation

**Figure 2:**
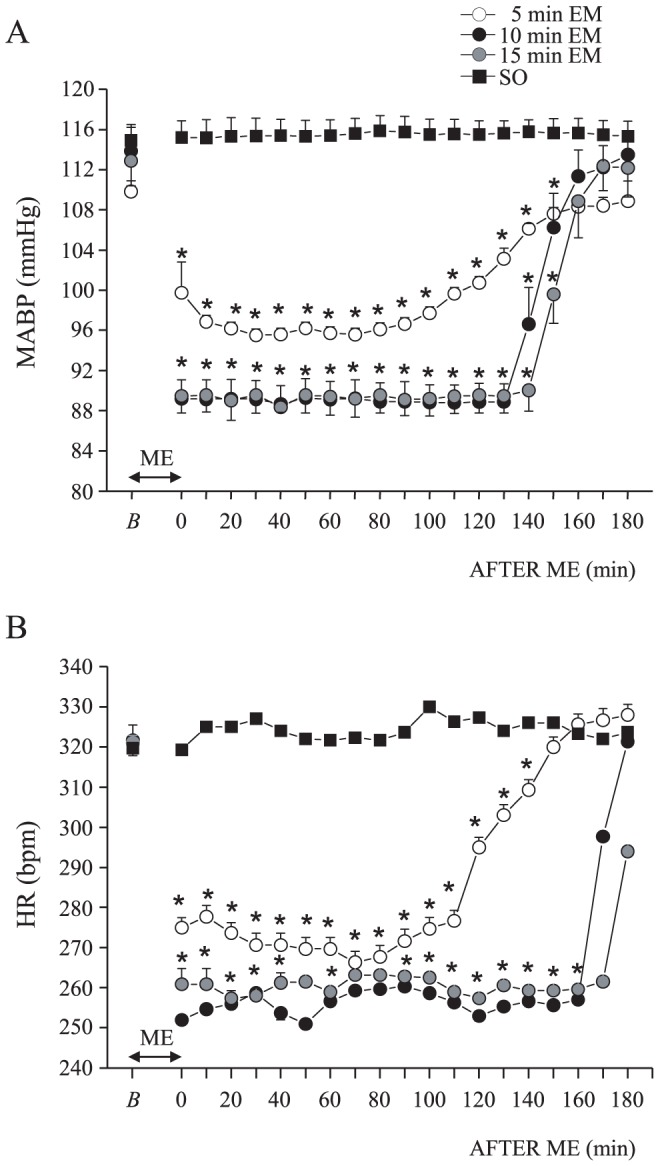
ME of different durations induced a reduction of MABP and HR. MABP(A) and HR (B) were recorded before (*B*) and after ME. ME of different durations induced an immediate significant reduction of MABP in comparison with the baseline value lasting for 140 minutes after 5 minutes ME (n = 5 rats, open circle) and for 160 minutes after 10 or 15 minutes ME (n = 5 rats, filled circle and n = 5 rats, grey circle respectively). In control rats (n = 5 rats, SO) MABP did not change for the whole observation period (filled square). HR significantly decreased immediately after removing ME of different durations. After 5 minutes ME, HR persisted significantly reduced up to 140 minutes (open circle), while after 10 (filled circle) or 15 minutes (grey circle) ME, HR was significantly decreased up to 160 minutes. In SO rats (filled square), HR was constant for the whole observation period. * Significantly different from the baseline value.

As previously shown [11], the cardiovascular response to ME consisted of an initial gradual reduction of MABP and HR during ME that persisted after ME (80 minutes) without recovering the initial value. We extended the observation time after ME. Fig. 2A shows the MABP values. After 5 minutes ME, MABP significantly declined from 109.90±1.07 mmHg to 99.82±3.06 mmHg immediately after ME (One way ANOVA for repeated measures, F_19.119_ = 267.40, p<0.0001, ηp = 1, post hoc test p<0.01); afterwards it remained stably reduced for about 90 minutes, and then gradually increased in the following 50 minutes remaining however still significantly reduced compared to the baseline value up to 140 minutes (106.20±0.52 mmHg, p<0.01). A similar response pattern was found for HR (○ in Fig. 2B). In fact, immediately after 5 minutes ME, HR significantly decreased from 321.67±3.82 bpm to 275.00±2.50 bpm (F_19,119_ = 401.20, p<0.0001, ηp = 0.923, post hoc test p<0.01) and then remained significantly reduced up to 140 minutes after ME (309.33±2.52, p<0.01 *vs* baseline value).

Rats subjected to 10 minutes ME presented, after ME, a significant reduction of MABP which decreased from 113.94±2.34 mmHg to 89.28±1.87 mmHg (F_19.99_ = 214.00, p<0.0001, ηp = 1, post hoc test p<0.01). MABP remained stably reduced for about 130 minutes, and then steeply increased in the following 30 minutes, remaining however still significantly reduced compared to the baseline value up to 160 minutes (111.4±2.62 mmHg, p<0.01). A similar response pattern was found for HR (• in Fig. 2B), which significantly diminished from 321.67±1.44 bpm to 252.00±1 bpm immediately after ME (F_19.99_ = 855.70, p<0.0001, ηp = 1, post hoc test p<0.01), and it persisted reduced up to 160 minutes after ME (at 160 minutes: 257.00±0.50 bpm, p<0.01 *vs* baseline value) and subsequently recovered the value observed in basal conditions. Increasing the time duration of ME to 15 minutes, the same trend as after 10 minutes ME was observed: MABP and HR decreased from the baseline value of 112.95±2.48 mmHg and 321.00±1.87 bpm respectively, to 89.56±1.71 mmHg (F_19.99_ = 215.40, p<0.0001, ηp = 1, post hoc test p<0.01) and 260.91±3.90 bpm (F_19.99_ = 214.00, ηp = 1, p<0.0001, post hoc test p<0.01) respectively. Afterwards both parameters persisted significantly reduced up to 160 minutes after ME (p<0.01 *vs* baseline value).

No changes of MABP and HR were observed in control rats (SO), which were only subjected to surgical procedures (▪ in Fig. 2A; Fig. 2B).

**Figure 3:**
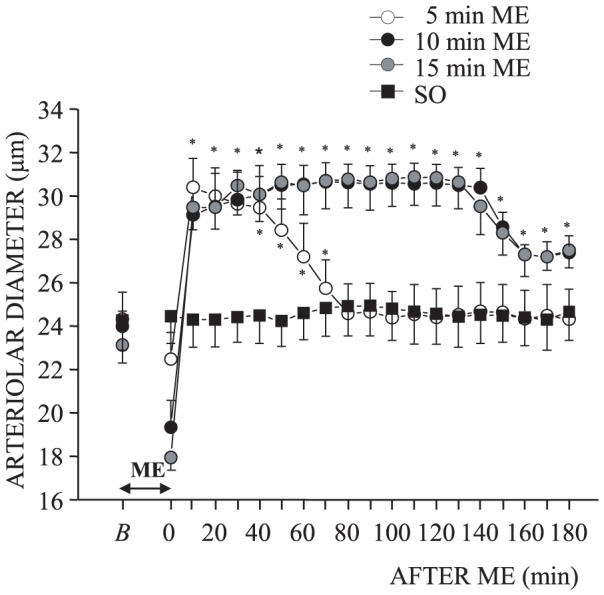
ME of different durations caused a biphasic response in order 2 pial arterioles. The values of the diameters of order 2 arterioles have been measured before and after ME. ME of different durations induced a simultaneous vasoconstriction followed by a vasodilation persisting for 70 minutes after 5 minutes ME (open circle) and for the whole after ME observation period (180 minutes) after 10 (filled circle) or 15 (grey circle) minutes ME. No changes in diameter values have been detected in SO rats, (filled square). * Significantly different from the baseline value.

Pial arterioles displayed a characteristic biphasic response, consisting in an initial vasoconstriction during ME, followed by a prolonged dilatation after ME. In particular order 2 arterioles during 5, 10 or 15 minutes ME, according to the time-duration of ME, exhibited a significant diameter decrease respect to the baseline value from 24.32±1.25 µm to 22.48±1.23 µm at the end of 5 minutes ME (F_19.99_ = 107.80, p<0.0001; ηp = 1, post hoc test, p<0.01; Fig. 3), from 23.99±0.71 µm to 19.34±1.24 µm at the end of 10 minutes ME (F_19,99_ = 107.80, p<0.0001; ηp = 1, post hoc test, p<0.01; Fig. 3) and from 23.13±0.83 µm to 17.95±0.56 µm at the end of 15 minutes ME (F_19.99_ = 265.40, p<0.0001; ηp = 1 post hoc test, p<0.01; Fig. 3). The animals subjected to 5 minutes ME showed, up to 40 minutes after ME, an increase of the arterioles diameter which then gradually decreased in the following 30 minutes, until reaching the baseline value (at 70 minutes: 25.74±1.32 µm, p<0.01 *vs* baseline value). Rats subjected to 10 or 15 minutes ME presented a vasodilation which persisted for the whole observation period after ME without achieving the baseline value (Fig. 3).

The vasoconstriction observed during 5, 10 or 15 minutes ME was accompanied by a significant reduction of capillary RBC velocity (0.12±0.02 mm/s, F_2.14_ = 69.06, p<0.0001; ηp = 0.95, post hoc test, p<0.01; 0.15±0.02 mm/s, F_2.14_ = 68.77, p<0.0001; ηp = 1, post hoc test, p<0.01; and 0.13±0.04 mm/s, F_2.14_ = 32.28, p<0.0001; ηp = 1, post hoc test, p<0.05, respectively) compared with the baseline values (0.19±0.02 mm/s, 0.21±0.01 mm/s and 0.20±0.03 mm/s, respectively). After ME the capillary RBC velocity increased, in particular in animals subjected to 5 minutes ME capillary RBC velocity was 0.28±0.02 mm/s up to 40 minutes after ME (p<0.01) and afterwards recovered the baseline value. Rats subjected to 10 or 15 minutes ME exhibited a capillary RBC velocity of 0.31±0.03 mm/s (p<0.01) and 0.32±0.03 mm/s (p<0.05), respectively for the whole observation period. Control rats (SO) did not exhibit any arteriolar diameter changes for the whole observation period and the capillary RBC velocity was stably 0.21±0.02 mm/s.

### The inhibition of nociceptors modifies the pial arterioles response during ME

**Figure 4:**
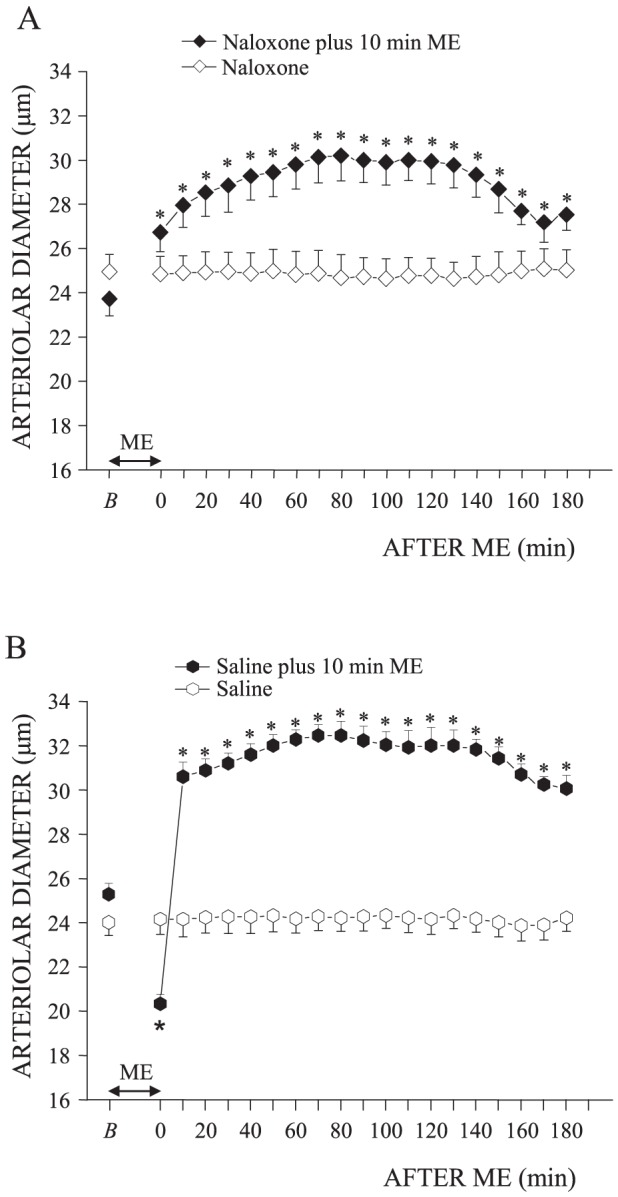
Nociceptors inhibition with naloxone abolished the vasocostriction during 10 minutes ME. (A) Naloxone was administered as described in Fig. 1B. Naloxone alone did not modify the diameter of order 2 arterioles (n = 4 rats, open rhomb), while, when associated with ME, it suppressed (n = 4 rats, filled rhomb) the constriction observed during ME, but did not affect the dilation observed after ME. (B) Saline administration (see Fig. 2B) did not influence the ME effects on pial arterioles diameter (n = 4 rats, filled hexagon), while, when applied alone, (n = 4 rats) it did not induce any changes in pial arterioles diameter (open hexagon). * Significantly different from the baseline value.

The intravenous administration of naloxone 10 minutes before 10 minutes ME did not affect the effects of ME on MABP and HR (Table 2), while it abolished the vasoconstriction concomitant with ME. In particular, the order 2 vessels mean diameter increased from 23.73±0.77 µm (baseline value, *B*) to 26.73±0.86 µm at the end of ME (One way ANOVA for repeated measures, F_19.119_ = 42.98, p<0.0001, ηp = 1, post hoc test, p<0.01 vs. baseline value; Fig. 4A). The vasodilation lasted for the whole period after ME and was accompanied by a significant increase of capillary RBC velocity (0.29±0.01 mm/s, paired t test, t = 7.086, df = 4, p = 0.0021) compared with the baseline value (0.20±0.03 mm/s). On the contrary, rats subjected to 10 minutes ME (Fig. 4A) and treated with saline intravenously administered 10 minutes before 10 minutes ME (Fig. 4B) exhibited the typical vasoconstriction followed by vasodilation accompanied by a significant reduction of capillary RBC velocity (0.13±0.02 mm/s, F_2.14_ = 55.75, p<0.0001; ηp = 0.97, post hoc test, p<0.05) in comparison with the baseline value (0.19±0.02 mm/s) during vasoconstriction, while a significant increase of capillary RBC velocity (0.30±0.03 mm/s, p<0.05) occurred during vasodilation. Also in these rats, the intravenous administration of saline did not affect the effects of ME on MABP and HR (Table 2). Finally, animals treated with naloxone alone did not exhibit changes in MABP, HR (Table 2) and in arteriolar diameter (Fig. 4A), as was the case for the rats treated with saline alone (Table 2; Fig. 4B).

**Table 2:** Effects of intravenous administration of naloxone or saline on MABP and HR.

	Naloxone +10 min ME		Saline +10 min ME		Naloxone			Saline	
	MABP (mmHg)	HR (beats/min)	MABP (mmHg)	HR (beats/min)		MABP (mmHg)	HR (beats/min)	MABP (mmHg)	HR (beats/min)
Baseline	113.07±1	319.00±0.71	116.85±1.57	330.50±1.20	Baseline	110.40±1.02	332.70±1.25	105.23±1.07	329.80±1.10
ME end	93.37±0.88	262.33±0.41	101.90±1.71	283.80±1.15	10 min	110.35±1.04	332.00±1.32	105.28±1.20	329.40±0.83
10 min	89.17±0.73	263.33±1.08	93.88±1.65	282.22±0.48	20 min	110.55±1.02	332.25±1.00	105.08±1.19	330.00±1.26
20 min	88.87±0.83	260.33±0.41	93.85±1.73	281.90±1.50	30 min	110.70±1.02	331.90±0.84	105.03±1.23	330.33±1.14
30 min	88.73±0.78	263.33±0.41	93.63±1.82	283.00±0.75	40 min	110.73±1.06	331.77±0.94	105.08±1.27	330.90±1.30
40 min	88.30±0.74	266.33±1.08	93.35±1.71	282.97±1.00	50 min	110.53±1.08	332.40±0.37	105.23±1.40	329.97±0.74
50 min	88.33±0.72	264.67±0.41	93.15±1.78	280.55±0.88	60 min	110.53±1.03	332.95±1.20	105.55±1.34	329.74±0.98
60 min	88.07±0.81	263.33±1.08	93.48±1.73	281.73±0.44	70 min	110.60±0.97	332.50±1.18	105.55±1.21	330.41±1.10
70 min	88.07±0.76	262.33±0.41	93.35±1.92	281.90±0.73	80 min	110.55±1.03	331.98±1.45	105.38±1.17	330.74±1.20
80 min	88.00±0.80	262.00±0.71	93.50±1.90	282.7 ±1.03	90 min	110.55±0.99	331.71±0.96	105.05±1.28	330.00±1.00
90 min	88.37±0.72	263.00±0.71	93.23±2.12	283.44±1.08	100 min	110.48±0.92	332.00±1.50	104.85±1.22	329.85±1.11
100 min	88.43±0.70	261.33±0.41	93.40±1.97	284.76±0.85	110 min	110.58±0.86	332.00±1.28	104.88±1.26	329.70±1.17
110 min	88.37±0.61	261.00±0.71	93.38±1.80	284.00±0.55	120 min	110.50±0.80	332.55±0.95	105.25±1.16	329.70±0.75
120 min	88.63±0.63	264.00±0.71	93.23±1.89	282.58±1.70	130 min	110.63±0.90	331.94±1.50	105.50±1.31	329.50±1.44
130 min	88.60±0.81	264.67±0.41	93.33±1.94	281.21±1.08	140 min	110.70±0.97	331.47±0.90	105.55±1.27	329.23±1.18
140 min	89.07±0.63	263.67±0.41	93.28±1.99	282.35±0.90	150 min	110.78±1.02	331.60±1.05	105.40±1.19	329.90±1.30
150 min	92.40±1.35	266.00±0.71	96.70±2.43	280.87±0.85	160 min	110.73±0.97	331.94±1.20	105.33±1.23	330.80±0.60
160 min	100.67±0.70	301.00±0.71	104.98±2.81	283.02±0.65	170 min	110.78±0.83	332.08±1.50	105.03±1.18	330.45±1.12
170 min	109.37±1.65	321.00±0.71	113.35±2.39	310.50±1.55	180 min	110.58±1.02	332.75±1.20	104.95±1.23	330.00±1.10
180 min	111.93±1.12	321.00±0.71	116.38±2.01	330.00 1.35	190 min	110.53±1.00	332.50±1.55	104.98±1.19	330.00±1.50

The values were recorded in the baseline condition, at the end of ME, and every 10 min during the after ME period in rats subjected to administration of naloxone or saline and 10 min ME. The value were recorded in the baseline condition and every 10 min for 190 min in rats subjected to the administration of naloxone or saline alone.

### Nitric oxide synthase inhibition affects the ME-induced effects on pial microcirculation

**Figure 5:**
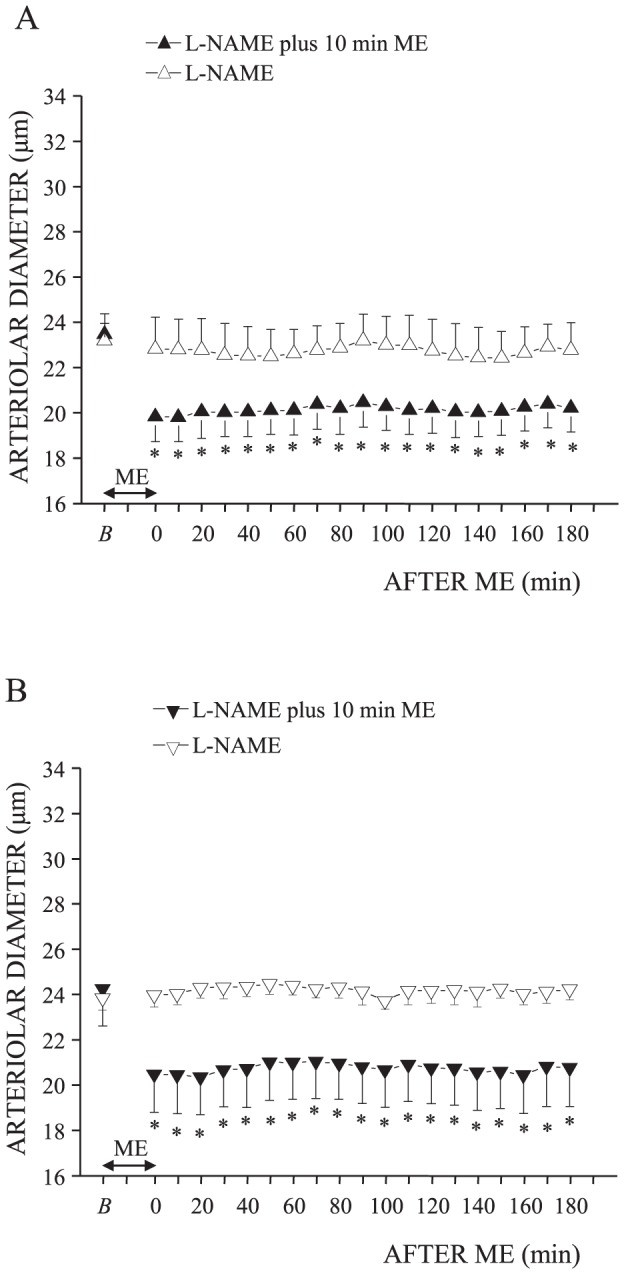
eNOS inhibition with L-NAME blocked the vasodilation induced by 10 minutes ME. Both i.v. infusion of L-NAME (n = 4 rats, see Fig. 1B) (A) and topical application of L-NAME (n = 4 rats, see Fig. 1C) (B) abolished the increase of the pial order 2 arterioles diameter (filled triangle) due to ME without affecting the constriction observed during ME. L-NAME alone (n = 4 rats, open triangle) did not induce any changes in the pial order 2 arterioles diameter. * Significantly different from the baseline value.

Local or intravenous administration of L-NAME for 10 minutes before 10 minutes ME did not affect the ME-induced response of MABP and HR (Table 3; Table 4), while they completely abolished the prolonged pial arterioles dilation observed after ME (Fig. 5A; Fig. 5B). In fact, after both L-NAME treatments, pial arterioles remained constricted for the entire period after ME without recovering the baseline value. The diameter of order 2 arterioles reduced from 26.75±1.06 µm to 22.60±1.10 µm (One way ANOVA for repeated measures, F_19.79_ = 43.14, p<0.0001, ηp = 1, post hoc test, p<0.01; Fig. 5A) in animals treated with L-NAME intravenously administered and from 28.48±1.90 µm to 24.10±1.97 µm (F_19.79_ = 49.04, p<0.0001, ηp = 1, post hoc test, p<0.01; Fig. 5B) in rats treated with L-NAME locally applied. Capillary RBC velocity significantly reduced after ME from 0.21±0.01 to 0.12±0.04 mm/s (paired t test, t = 5.034, df = 4, p = 0.0073) and from 0.19±0.03 to 0.10±0.02 mm/s (paired t test, t = 5.416, df = 4, p = 0.0056) in rats treated with intravenous and local administration of L-NAME, respectively.

**Table 3:** Effects of local administration of L-NAME on MABP and HR.

	L-NAME +10 min ME		L-NAME		
	MABP (mmHg)	HR (beats/min)		MABP (mmHg)	HR (beats/min)
Baseline	108.90±1.53	341.67±0.88	Baseline	105.38±1.98	333.00±1.50
ME end	92.98±1.61	298.33±0.88	10 min	105.63±1.94	332.85±0.99
10 min	91.48±1.43	296.33±0.88	20 min	106.00±1.94	332.33±0.85
20 min	91.55±1.48	294.67±0.33	30 min	106.13±1.94	331.98±0.75
30 min	91.73±1.43	297.67±0.33	40 min	106.08±2.24	331.37±1.40
40 min	92.05±1.42	297.67±0.33	50 min	105.90±2.13	332.39±0.95
50 min	92.45±1.44	297.33±0.33	60 min	105.98±2.02	332.99±1.40
60 min	92.58±1.51	300.00±0.58	70 min	106.03±1.89	332.50±1.75
70 min	92.48±1.47	301.67±0.33	80 min	106.25±2.01	332.25±1.50
80 min	91.88±1.49	302.67±0.33	90 min	105.78±1.87	332.40±0.98
90 min	91.70±1.40	297.33±0.67	100 min	105.53±1.88	333.55±1.10
100 min	91.88±1.39	296.33±0.88	110 min	105.75±2.11	333.99±1.70
110 min	91.95±1.39	294.67±0.33	120 min	105.90±2.02	333.00±1.23
120 min	92.25±1.51	294.67±0.33	130 min	105.80±1.98	333.58±1.35
130 min	92.40±1.53	296.33±0.88	140 min	105.50±1.94	332.80±1.50
140 min	94.80±1.44	298.67±0.33	150 min	105.75±2.05	333.12±0.40
150 min	101.83±1.44	298.00±0.58	160 min	105.80±1.90	333.92±0.70
160 min	106.43±1.54	311.67±0.33	170 min	105.75±1.88	332.76±0.87
170 min	108.78±1.43	341.00±0.58	180 min	105.75±1.84	332.40±0.93
180 min	108.88±1.57	339.00±0.58	190 min	105.85±1.88	333.00±1.25

The values were recorded in the baseline condition, at the end of 10 min ME, and every 10 min during the after ME period in rats subjected to local administration of L-NAME and 10 min ME. The value were recorded in the baseline condition and every 10 min for 190 min in rats only subjected to local administration of L-NAME.

**Table 4:** Effects of i.v. administration of L-NAME on MABP and HR.

	L-NAME +10 min ME		L-NAME		
	MABP (mmHg)	HR (beats/min)		MABP (mmHg)	HR (beats/min)
Baseline	108.90±1.53	336.67±1.08	Baseline	107.75±1.08	328.70±1.30
ME end	92.98±1.61	283.00±0.71	10 min	107.80±0.97	329.00±0.55
10 min	91.48±1.43	281.00±0.71	20 min	107.68±1.10	329.50±1.20
20 min	91.55±1.48	283.67±0.41	30 min	108.18±0.91	329.88±0.75
30 min	91.73±1.43	285.67±0.82	40 min	108.48±0.86	328.40±1.35
40 min	92.05±1.42	282.67±0.41	50 min	108.58±1.04	328.12±1.27
50 min	92.45±1.44	287.33±0.41	60 min	108.38±1.13	328.54±1.22
60 min	92.58±1.51	291.00±0.71	70 min	108.45±1.39	329.00±1.08
70 min	92.48±1.47	291.67±0.41	80 min	108.48±1.36	329.50±1.24
80 min	91.88±1.49	289.00±0.71	90 min	108.25±1.27	329.00±1.15
90 min	91.70±1.40	288.00±0.71	100 min	108.33±1.13	328.55±1.20
100 min	91.88±1.39	285.67±0.41	110 min	108.13±0.94	328.74±1.68
110 min	91.95±1.39	283.67±0.82	120 min	108.15±0.86	328.93±0.87
120 min	92.25±1.51	282.00±1.23	130 min	108.33±0.93	329.43±1.72
130 min	92.40±1.53	284.33±0.41	140 min	108.45±0.97	329.84±1.50
140 min	94.80±1.44	285.33±0.41	150 min	108.45±0.98	328.95±1.08
150 min	101.83±1.44	291.00±0.71	160 min	108.43±0.92	328.78±0.95
160 min	106.3±1.54	311.00±0.71	170 min	108.43±0.96	328.37±0.67
170 min	108.78±1.43	334.33±1.48	180 min	108.00±0.96	328.69±1.35
180 min	108.88±1.57	336.00±0.71	190 min	108.23±1.13	328.90±1.10

The values were recorded in the baseline condition, at the end of 10 min ME, and every 10 min during the after ME period in rats subjected to i.v. administration of L-NAME and 10 min ME. The value were recorded in the baseline condition and every 10 min for 190 min in rats only subjected to i.v. administration of L-NAME.

L-NAME intravenously or locally administered in rats not subjected to 10 minutes ME did not cause changes of the parameters evaluated throughout all the observation period (Table 3; Table 4; Fig. 5A; Fig. 5B).

Interestingly, the vasodilation observed after 10 minutes ME (28±3.2% of the baseline value) did not represent the maximal dilation of order 2 arterioles, because the local application of 100 µM acetylcholine induced a vasodilation of 41.5±2.7% of the baseline value in sham-operated animals (data not shown) as previously observed by Oriji [23].

### Effects of ME on NO synthase expression

**Figure 6:**
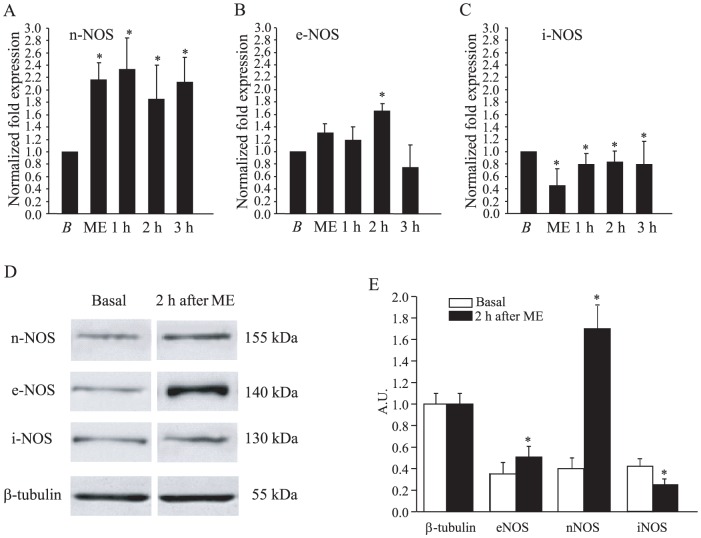
The expression of nNOS and e NOS was increased 2 hours after 10 minutes ME. (A) The gene coding for nNOS was significantly more expressed at all the times considered with respect to the baseline value (B). The expression of the gene coding for eNOS was significantly more expressed at 2 hours after ME with respect to all the other time points considered (C). The gene coding for iNOS was significantly less expressed at all times after ME with respect to the baseline value. (D) Western blotting performed with brain samples from rats sacrificed 2 hours after ME shows that, at this time, the blot for nNOS and e NOS was more positive compared to the ones obtained in baseline conditions (upper blots), whereas both the iNOS expression and the β-tubulin expression (lower blots) were comparable at the two times considered. (E) Histograms showing the ratio of the optical density between β-tubulin and eNOS, nNOS and iNOS calculated for the baseline condition and 2 hours after ME. * Significantly different from the baseline value. Abbreviations: A.U. arbitrary unit.

The results just reported are consistent with the notion that the vasodilation observed after ME is mediated by a mechanism involving nitric oxide (NO). Therefore, we evaluated the gene expression of the three isoforms of NOS during the baseline period, at the end of ME and 1, 2 and 3 hours after ME. The results obtained by qRT-PCR assay are summarized in Fig. 6. The histograms show the ratio between the transcript level of each target gene and the G3PDH gene expression level normalized to the control sample (baseline condition, *B* in Fig. 6A, 6B, 6C). Fig. 6A shows that ME was accompanied by a significant increase in the expression of nNOS which persisted for the whole observation period (baseline value: 1.00±0.01, at the end of ME: 1.38±0.21; 1 hour after ME: 1.22±0.13; 2 hours after ME: 1.49±0.13; 3 hours after ME: 1.40±0.10; One Way ANOVA, F_4.47_ = 3.274, p = 0.019; post hoc test, ηp = 0.92, p<0.05, for all the times considered compared to the baseline value). The expression of eNOS gene (Fig. 6B) was markedly increased 2 hours after ME (baseline value: 1±0.02, at the end of ME: 1.10±0.09; 1 hour after ME: 1.10±0.17; 2 hours after ME: 1.71±0.25; 3 hours after ME: 1.32±0.23, F_4.47_ = 2.68, p = 0.04, ηp = 0.93, post hoc test, p<0.05, 2 hours after ME vs. baseline value).

Interestingly, the expression of iNOS gene was significantly reduced by ME (Fig. 6C; baseline value: 1.00±0.07; at the end of ME: 0.67±0.08; 1 hour after ME: 0.79±0.09; 2 hours after ME: 0.83±0.07; 3 hours after ME: 0.790±0.066, F_4.59_ = 5.73, p = 0.001, post hoc test, p<0.05 for all the times considered compared to the baseline value).

Finally, we analyzed the protein expression of the three NOS isoforms during the baseline period and 2 hours after ME. As shown in Fig. 6D and Fig. 6E, at 2 hours after ME the synthesis of nNOS (0.52±0.10 A.U.) was significantly increased in comparison with the value obtained in control rats (0.38±0.06 A.U., unpaired t test, t = 3.202, df = 8, p = 0.0126) as well as the eNOS synthesis (at 2 hours after ME: 1.70±0.20 AU, control value: 0.40±0.10 AU, unpaired t test, t = 20.37, df = 8, p<0.0001), while the iNOS synthesis was significantly decreased (at 2 hours after ME: 0.25±0.08 A.U.; control value: 0.43±0.07 A.U., unpaired t test, t = 6.020, df = 8, p = 0.0003).

These data suggest that the vasodilation observed immediately after ME was supported by a release of NO from neuronal cells; afterwards (2 hours after ME) NO release was mainly due to endothelial cells activity, and neuronal cells continued to sustain NO release until 3 hours after ME.

## Discussion

In a previous paper [11] we noted that in the anesthetized rat, a passive ME of 5 to 15 minutes caused a prolonged reduction of MABP and HR associated with a biphasic pial microcirculation response, which were abolished by trigeminal nerve cutting. In that study, the follow-up after ME (80 minutes) was insufficient to determine the actual duration of these effects. In the present study we extended the follow-up by prolonging the observation time to 3 hours, thus being able to determine the full time course of the hypotensive and bradycardic responses until recovery. Conversely, the dilation of pial arterioles did not recover the basal value within 3 hours.

The duration of ME seemed to depend on both the time course and the extent of the responses. In fact, 5 minutes ME determined a hypotensive and bradycardic effect that was less prolonged and somewhat less pronounced, compared to 10–15 minutes ME, that, by converse, had essentially identical curves and much steeper recoveries (see Fig. 2).

It has been known for some time that trigeminal sensory stimulation may elicit cardiovascular depressor responses; however, to our knowledge, in no instance, effects as prolonged as those observed here (of two hours or more) have been reported [20], [24], [5].

The responses of pial microcirculation to ME were more complex and differed in various aspects from those of blood pressure and heart rate. In fact, concomitantly with the reduction of MABP and HR during ME, the pial arterioles (unexpectedly) constricted, as previously observed [11], whereas following ME, a prolonged overshooting vasodilation occurred with a time course that depended on the duration of ME. For 5 minutes of ME the vasodilator effect persisted for about 40 minutes and then gradually subsided in the following 30 minutes, whereas for longer ME (10–15 minutes) the vasodilation persisted for the whole observation period (180 minutes), although it was less pronounced in the last 40 minutes. The vasoconstriction was always accompanied by a significant reduction of the capillary RBC velocity while the vasodilation induced an important increase in the capillary RBC velocity.

This biphasic response of pial microcirculation to ME is somewhat surprising and deserves consideration, since, a systemic blood pressure decrease is usually accompanied by a vasodilation of pial arterioles. However in our model the reduction of MABP during ME was associated with an initial constriction of pial arterioles followed by a prolonged dilation after ME. Interestingly, these two responses seem to be mediated by different mechanisms. In fact, the initial vasoconstriction (but not the subsequent vasodilatation) was found to be abolished by naloxone, while the subsequent prolonged vasodilatation (but not the initial vasoconstriction) was abolished after inhibition of NO synthase by L-NAME. Since it has been shown that naloxone can directly modulate the activation of the trigeminal neurons by inhibiting specific opioid receptors [25], we suggest that the vasoconstrictor effect in pial microcirculation during ME was elicited by an opioid receptor-mediated mechanism triggered by trigeminal discharge. However, while opioid-mediated mechanisms appear to play a major role in the pial arterioles constriction response upon trigeminal stimulation, they do not exert a similar role on systemic circulation since naloxone did not affect MABP and HR decrease.

The pial arterioles dilation concomitant with the reduction of MABP and HR after ME may represent a compensatory autoregulatory response of pial microcirculation secondary to changes in perfusion pressure, as observed in several animal models [26], [27]. However, a few aspects seem to suggest that pial vasodilation is not simply an autoregulatory response secondary to the hypotensive effect due to ME. Interestingly, the pial vasodilatory response appears to be at least in part dissociated from the hypotensive response, as observed by comparing their time-course for 5 minutes ME: in fact, while MABP remained stably low for about 100 minutes, maximal pial vasodilatation persisted for only 40 minutes, recovering to basal values in the following 40 minutes at a time where the hypotensive effect was still fully present. By prolonging the ME duration (10 or 15 minutes), MABP and HR recovered the basal value within 150 minutes and 160 minutes respectively, while the pial arterioles persisted significantly dilated for the whole observation period.

Little is known about the mechanisms underlying the dilation of cerebral vessels associated with trigeminal stimulation. Some authors observed that a powerful and differentiated activation of sympathetic system occurs within seconds after the initiation of a trigeminocardiac reflex which leads to an increase in regional cerebral blood flow with no changes in cerebral metabolic rate of oxygen or glucose [28]. Lindauer and co-workers suggested that an increase in neuronal activity due to trigeminal nerve stimulation may be correlated with neuronal nitric oxide release affecting the arterioles adjacent to the activated neuronal pool [29]. Our results show an increase of the nNOS expression during and after ME, which did not result in a vasodilation during ME. According to Gong et al. [30] and Moncada et al., [31], NO released by nNOS probably maintains the integrity and the homeostasis of neuronal cells initially and only successively (after ME) it contributes to the vasodilation. On the other hand, the opioid-mediated effects leading to vasoconstriction may overcome the vasodilatory effect of NO during ME. In the after ME period, the pial vasodilation was sustained not only by the activity of nNOS which was highly expressed for the whole observation period, but also by eNOS activity, mainly at 2 hours. In fact, the real time qRT-PCR assay on samples of the parietal cortex which receives the main trigeminal afferents, showed a significant increase of the expression of the gene coding for nNOS during and after ME and a progressive increase in the expression of the gene coding for eNOS, statistically significant at 2 hours after ME when compared to the baseline condition. Moreover, western blotting analysis confirmed the increase in expression of the protein nNOS and eNOS at 2 hours after ME. The expression of the gene coding for iNOS was significantly reduced during and after ME for the whole observation period and at 2 hours after ME the protein expression was also reduced with respect to the baseline condition, indicating that ME is a non-invasive method devoid of harmful (inflammatory) side effects [13].

In conclusion, the effects of ME on cardiovascular parameters appear to be complex, because they involve different mechanisms. Opioid receptor activation during ME and NO release by neuronal and endothelial vascular cells after ME affect pial microcirculation, whereas the systemic responses causing decrease in MABP and HR seems to be related to other mechanisms probably involving activation of vagal efferents to the heart and modulation of vasomotor neuron discharge on peripheral circulation. It is worth noting that the decrease in arterial blood pressure is not accompanied by an increase in heart rate. Further investigation carried out on the brainstem trigeminal and vagal nuclei could elucidate this issue.

## Supporting Information

S1 File (PPT)
