# Supplementary material for: Trigeminocardiac Reflex by Mandibular Extension on Rat Pial Microcirculation: Role of Nitric Oxide
Source: PLoS One. 2014 Dec 31;9(12):e115767. doi: 10.1371/journal.pone.0115767 (PMC4281058; doi:10.1371/journal.pone.0115767)
Supplement: S1 File — (PPT) [file pone.0115767.s001.ppt]

## Slide 1
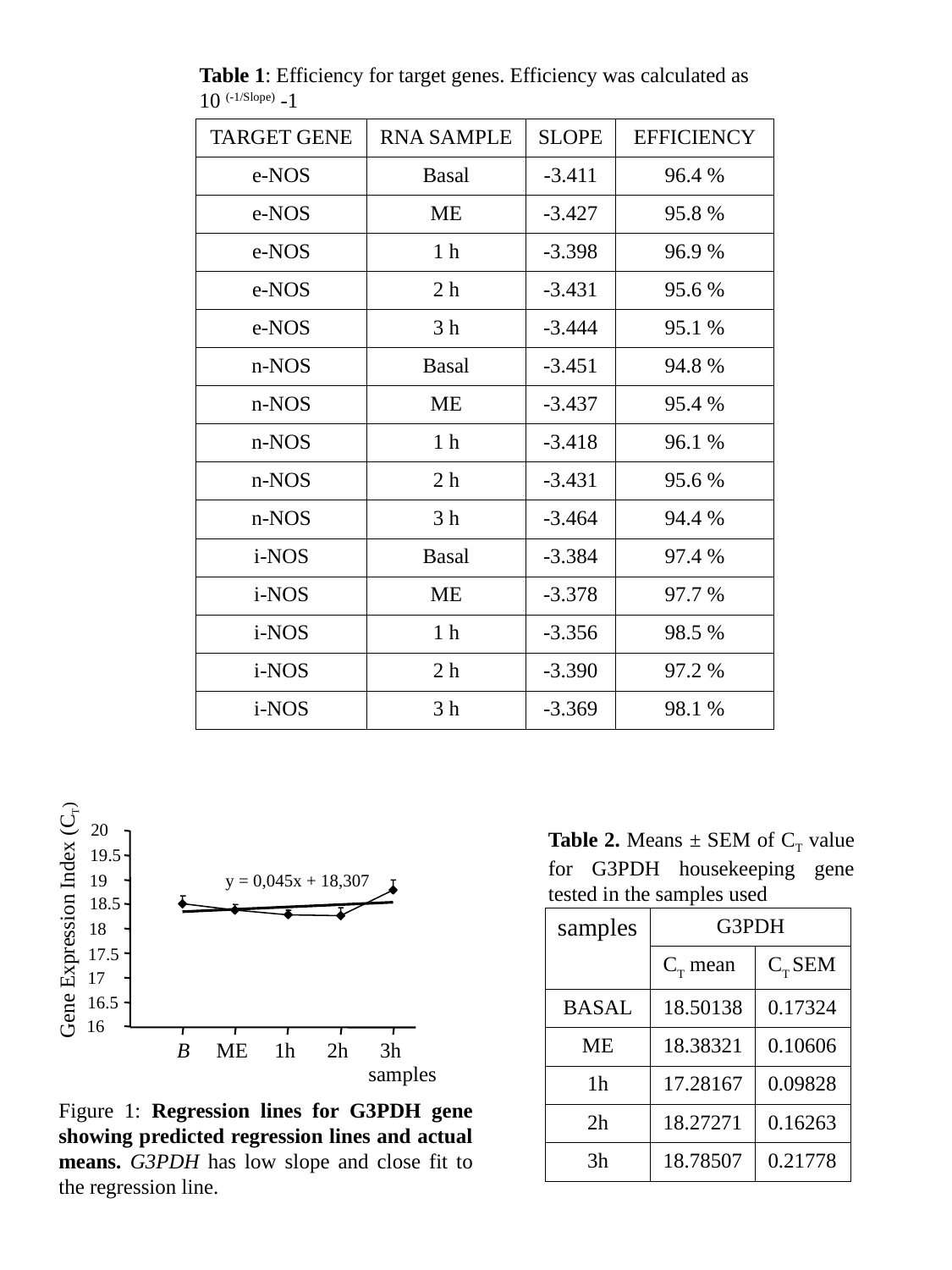

Table 1: Efficiency for target genes. Efficiency was calculated as 10 (-1/Slope) -1
| TARGET GENE | RNA SAMPLE | SLOPE | EFFICIENCY |
| --- | --- | --- | --- |
| e-NOS | Basal | -3.411 | 96.4 % |
| e-NOS | ME | -3.427 | 95.8 % |
| e-NOS | 1 h | -3.398 | 96.9 % |
| e-NOS | 2 h | -3.431 | 95.6 % |
| e-NOS | 3 h | -3.444 | 95.1 % |
| n-NOS | Basal | -3.451 | 94.8 % |
| n-NOS | ME | -3.437 | 95.4 % |
| n-NOS | 1 h | -3.418 | 96.1 % |
| n-NOS | 2 h | -3.431 | 95.6 % |
| n-NOS | 3 h | -3.464 | 94.4 % |
| i-NOS | Basal | -3.384 | 97.4 % |
| i-NOS | ME | -3.378 | 97.7 % |
| i-NOS | 1 h | -3.356 | 98.5 % |
| i-NOS | 2 h | -3.390 | 97.2 % |
| i-NOS | 3 h | -3.369 | 98.1 % |
20
Table 2. Means ± SEM of CT value for G3PDH housekeeping gene tested in the samples used
19.5
19
y = 0,045x + 18,307
18.5
Gene Expression Index (CT)
| samples | G3PDH | |
| --- | --- | --- |
| | CT mean | CT SEM |
| BASAL | 18.50138 | 0.17324 |
| ME | 18.38321 | 0.10606 |
| 1h | 17.28167 | 0.09828 |
| 2h | 18.27271 | 0.16263 |
| 3h | 18.78507 | 0.21778 |
18
17.5
17
16.5
16
B ME 1h 2h 3h
samples
Figure 1: Regression lines for G3PDH gene showing predicted regression lines and actual means. G3PDH has low slope and close fit to the regression line.
